# Improved X-ray computed tomography reconstruction of the largest fragment of the Antikythera Mechanism, an ancient Greek astronomical calculator

**DOI:** 10.1371/journal.pone.0207430

**Published:** 2018-11-09

**Authors:** Ashkan Pakzad, Francesco Iacoviello, Andrew Ramsey, Robert Speller, Jennifer Griffiths, Tony Freeth, Adam Gibson

**Affiliations:** 1 Department of Medical Physics and Biomedical Engineering, UCL, London, United Kingdom; 2 Electrochemical Innovation Lab, Department of Chemical Engineering, UCL, London, United Kingdom; 3 Nikon Metrology Inc, Michigan, United States of America; 4 Arena Centre for Research-Based Education, UCL, London, United Kingdom; 5 Department of Mechanical Engineering, UCL, London, United Kingdom; Institute for Anthropological Research, CROATIA

## Abstract

The Antikythera Mechanism is an extraordinarily complex ancient Greek astronomical calculating device whose mode of operation is now relatively well understood particularly since imaging studies in 2005 revealed gears and inscriptions which were previously illegible. Unfortunately, the highest resolution X-ray computed tomography image of the largest fragment had some errors which meant that the reconstructed images were not as clear as had been expected. Here, the original X-ray data have been reanalysed and reconstructed. The new X-ray computed tomography images have improved contrast and resolution, leading to better clarity and legibility. The improvement in image quality is characterised and some examples of writing on the Mechanism which can now be read with increased confidence are given.

## Introduction

The Antikythera Mechanism is a 2000-year-old device which used at least 30 bronze gears to predict astronomical and social events including eclipses, phases of the moon, positions of the planets in the sky and even the dates of the Olympic and other games. It was recovered from a shipwreck in 1900–1 and consists of 82 corroded and calcified fragments. Over time, photographs and radiographs led to an improved understanding of the Mechanism [1–3] until a new set of high resolution reflectance transform imaging (RTI) and X-ray computed tomography (CT) images were acquired in 2005 [4]. Analysis of these images led to new discoveries about the inscriptions, construction and gearing of the Mechanism [4–6].

The CT system used was made by X-Tek Systems Ltd (UK) (now Nikon Metrology): a *BladeRunner* with an experimental 450 kV micro-focus source, which was shipped from the UK to the National Archaeological Museum in Athens for the 2005 study [7]. Some hardware and software problems were reported during the two weeks of imaging, but these did not generally affect the resulting image quality.

Six different CT scans were taken of the largest fragment, Fragment A, with different energies and magnifications. The final scan, Scan 6, used a lower X-ray energy source (225 kV rather than 275 kV and 366 kV) that allowed the sample to get closer to the source, thereby providing greater magnification. The increased magnification factor meant that the pixel size at the centre of rotation was intended to be 54.2 μm rather than 101.0 μm for the other scans. Unfortunately, reconstructions of scan 6 were disappointing. In 2014, Freeth said, “The highest resolution scan of Fragment A was seriously compromised by a technical problem during data acquisition, whereby about 27 projections (compared to 2957 successfully acquired projections) failed to record. There is also evidence that the fragment moved during the scan. Attempts to rectify these problems have only been partially successful” [6]. Images from these partially successful reconstructions have been previously published (for example Fig 23 in [5] and Fig 5E in [6]).

We have reanalysed scan 6 to examine the two possibilities described above: movement of the fragment and missing projections. We have corrected the number of missing projections and reconstructed the resulting X-ray CT volume which shows the interior of Fragment A with enhanced clarity and resolution. We report on some modest improvements in the legibility of the text.

## Methods

### X-ray computed tomography

Here we give a summary of the X-ray process used to examine the fragments of the Antikythera Mechanism at the National Archaeological Museum in Athens in 2005 [4]. A more detailed description of the techniques can be found in supplementary *S1 Note: X-ray Techniques*.

**Fig 1:**
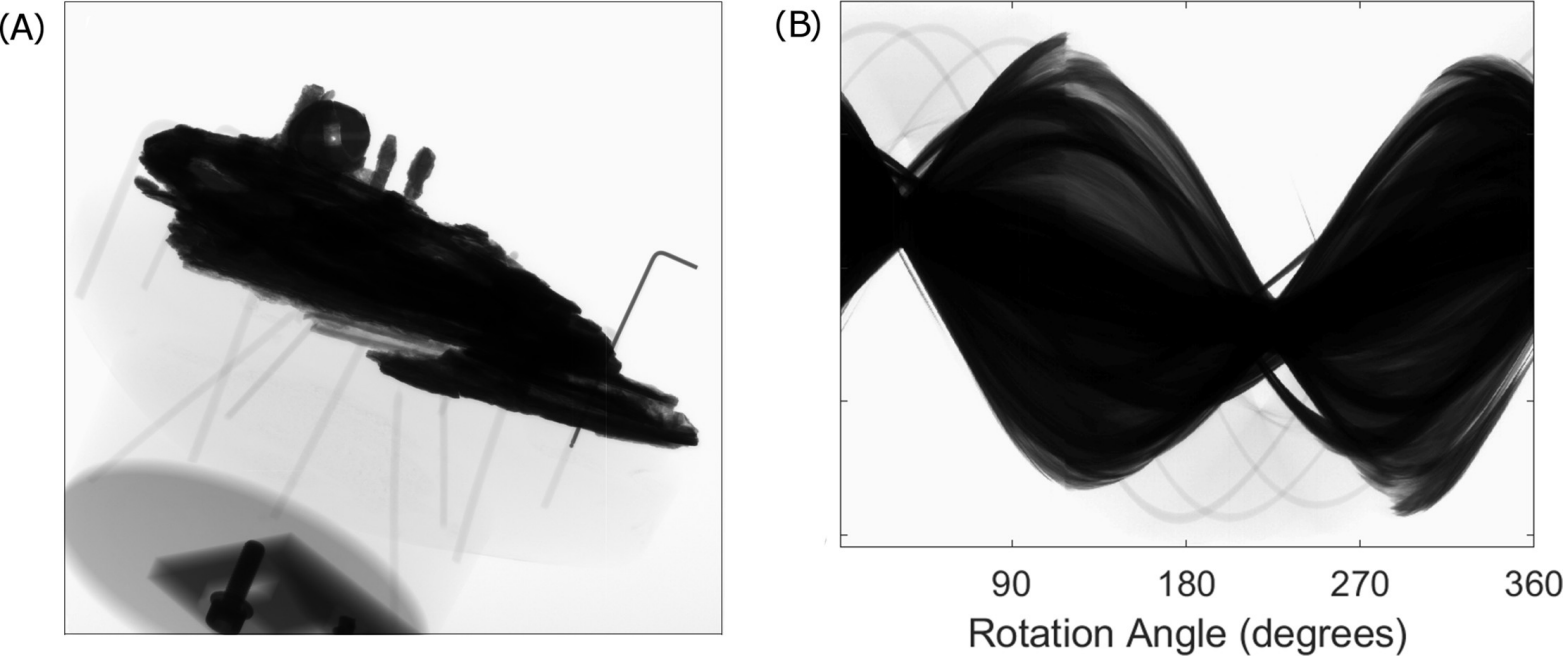
An example of (A) a projection and (B) a sinogram of the Antikythera Mechanism. In 1(A), the Mechanism can be seen in the top half of the image, along with an Allen key which was added as a known target and support structures. The Allen key and support structures are responsible for the faint, narrow lines in the sinogram (1(B)).

X-ray computed tomography is a long-standing medical imaging technique [8] which is now widely used in other areas of science, including heritage [9]. A series of planar X-ray images (known as “projections”; see Fig 1A) are taken as the object rotates relative to the X-ray source and detector. The X-ray CT carried out by X-Tek Systems employed a prototype X-ray machine, the *Bladerunner*. At 450 kV, this was the most powerful X-ray CT machine available, developed specifically to have sufficient X power to penetrate Fragment A of the Antikythera Mechanism at all angles—a prerequisite of the X-ray CT process. In medical imaging, the source and detector rotate around the patient, but the *BladeRunner* was a self-contained cabinet in which the source and detector are stationary and the object rotates. The projections were recorded on a Perkin Elmer flat panel detector and saved as TIFF images.

### A scan 6

For an X-ray CT scan, many projections are required; scan 6 of Fragment A consists of 3052 projections, including some oversampling to include more than a complete 360° rotation. A projection close to the beginning of the scan was matched to the corresponding projection near the end of the scan to determine a whole rotation of 360°. This resulted in 2957 projections. 25 missing projections were identified, as described below, and inserted in to the sequence at the appropriate positions and the whole set of projections were then renumbered as Proj0001 to Proj2982. This numbering system after correction is used throughout this paper.

The projections were gathered while the sample rotated continuously. This was because the Antikythera fragments are extremely fragile and X-Tek Systems advised that, if the X-ray machine were programmed to stop and start again for over 3,000 projections, the resultant vibration might damage the fragments.

Each projection image was corrected using dark and bright field reference images in order to reduce noise. These corrected images were reconstructed using the well-known Feldkamp, Davis and Kress (FDK) filtered backprojection algorithm [10] implemented in Nikon CTPro3D version XT5.1.3.1 (Nikon Metrology, Tring, UK). The FDK algorithm filters then backprojects the values from every projection image through the volume to calculate the X-ray linear attenuation values at the position of each volume element (voxel) within the reconstructed volume. These constitute the (floating-point) grey values within the CT volume.

### Movement of the fragment during the scan

Soon after the data were gathered, an initial volume reconstruction from the data was attempted by X-Tek Systems. This was unsuccessful in creating a meaningful volume: for example, features of the scan that should be circular had a twisted spiral appearance. It was hypothesised that the degraded reconstruction might have resulted from the movement of the sample during the scan. However, projections 360° apart were examined and found to be identical except for random noise, and so we conclude that the sample did not move during data acquisition.

### Missing projections

After further research, it was realized that some of the projections were missing. By stepping through the projections in sequence, it was possible to determine a segment of data between Proj0936 and Proj0972 where a series of projections at irregular intervals were missing: for example, there might be a gap of six missing projections, followed by another gap with one missing projection and then two missing projections etc. It appears that the computer failed to record some projections in this segment.

The determination of exactly where the missing projections occurred was aided by viewing the data as a sinogram (see Fig 1B). A single horizontal line through a projection represents a single slice through the object. To generate a sinogram, the same horizontal slice is identified in each projection and displayed vertically in sequence with the same slice taken from every other projection. The sinogram then shows how a single horizontal slice through the object varies with angle.

**Fig 2:**
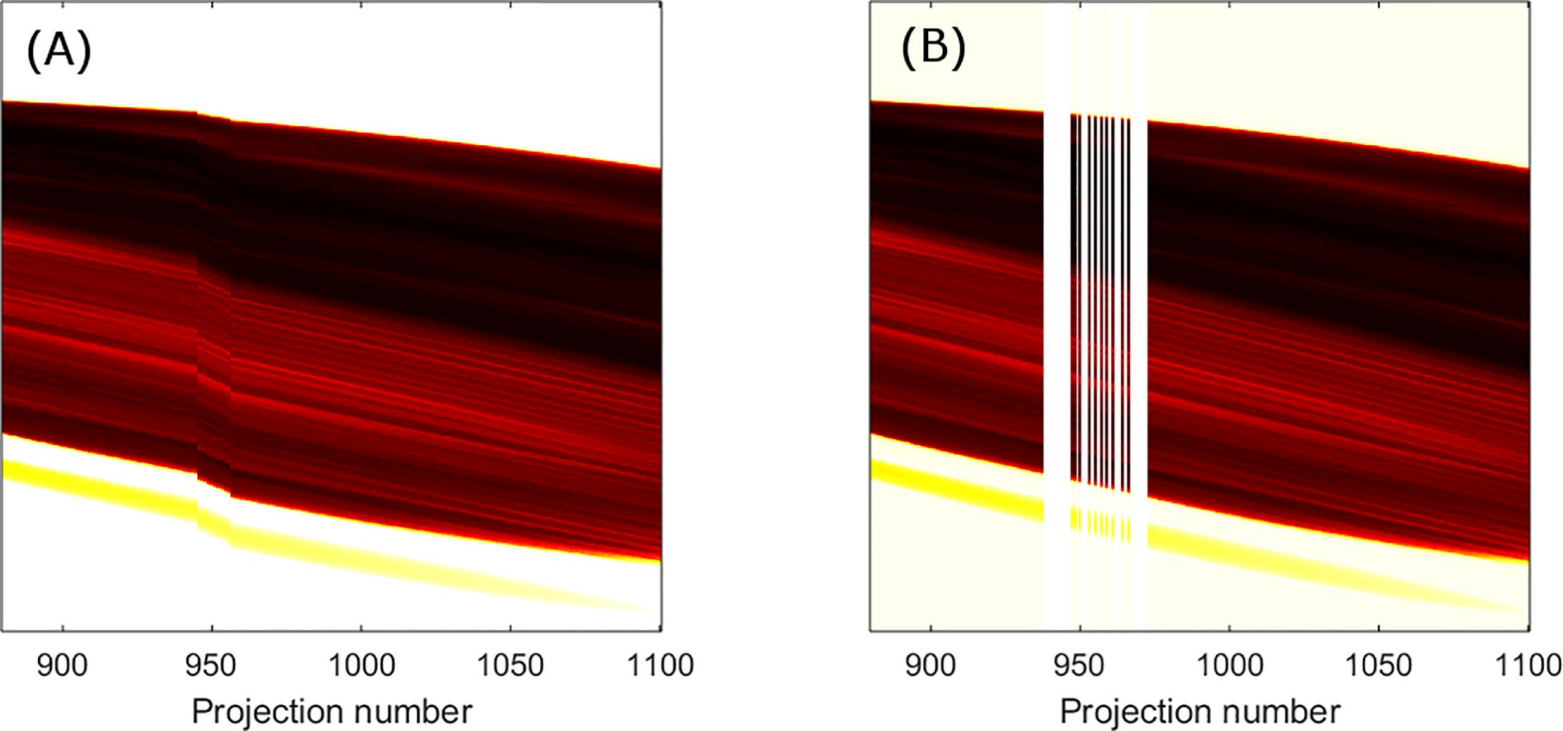
Magnified view of sinogram, showing (A) steps due to missing projection and (B) the flattened sinogram after correction for missing projections.

In Fig 1, a discontinuity can be seen in the sinogram at around 120° due to the missing projections. Using this method, it was also determined that there were no repeated projections. If these had occurred, they would have shown up in Fig 2, which illustrates the sinogram: missing or duplicated projections would cause a discontinuity in the sinogram; none were apparent.

**Fig 3:**
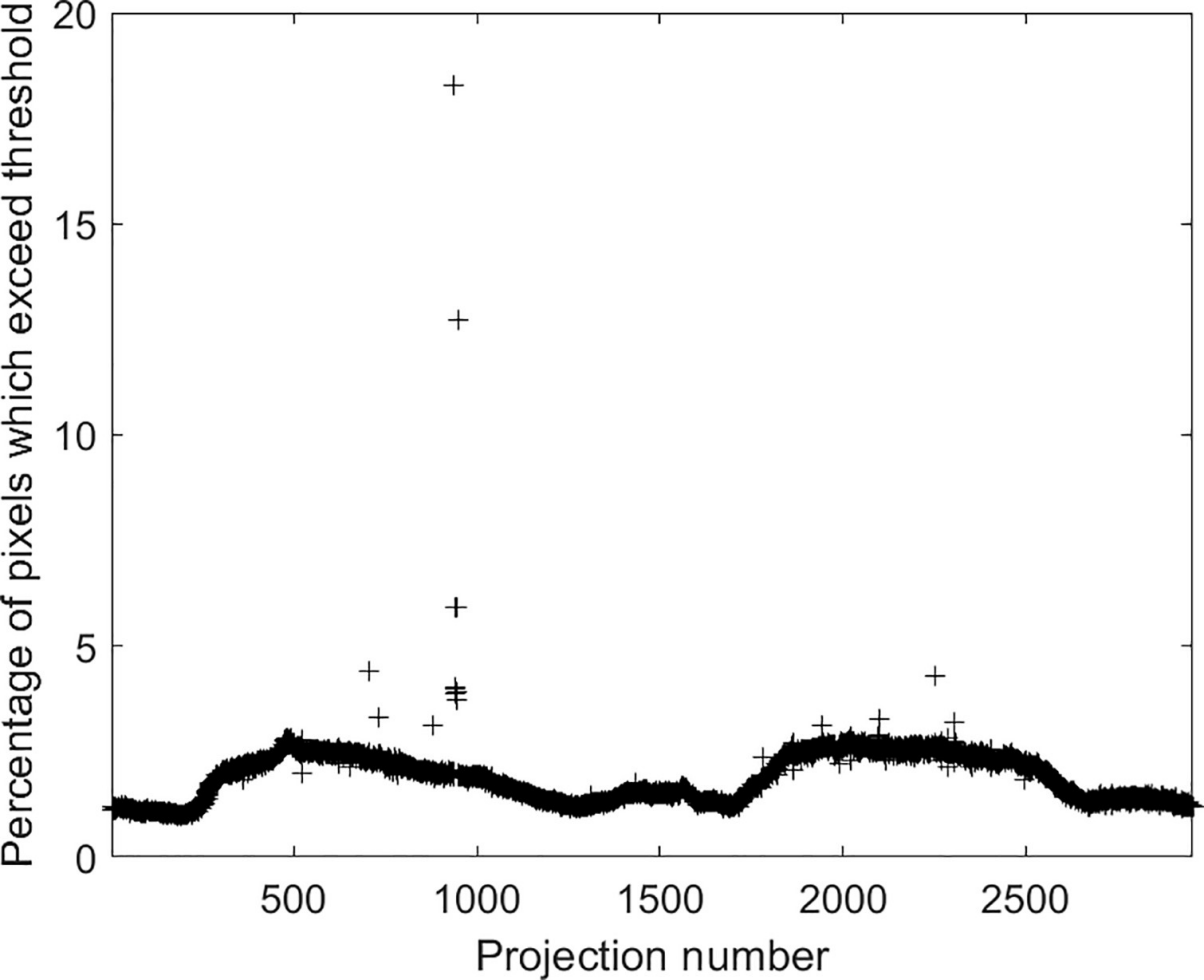
Percentage of pixels that exceed a threshold of mean + 5.3 standard deviations. A cluster of outliers can be seen around projection 950.

Missing projections were identified using a two-step process. First, the steps in the sequence where missing projections occurred were identified from the sinogram, and second, the number of missing projections at each step was found. This was done by subtracting neighbouring projections from each other and calculating the mean and standard deviations of the pixels making up the difference images. The Bonferroni correction [11] gave a threshold of 5.3 standard deviations for a statistically significant deviation. Fig 3 shows the percentage of pixels that exceed mean ± 5.3 standard deviations for the difference between adjacent projections, plotted against projection number. Across the 2957 projections, most difference images show <3% of pixels exceeding this threshold, the number changing as the fragment rotates (and showing a reflection about the midway point as the fragment rotates by 180°). However, in some areas, the number of outlying pixels substantially exceeds the background number, suggesting that the difference between neighbouring projections is greater than expected. Moreover, when this occurs once in the pattern (e.g. around projection 950), it is likely to be inherent in the data collection and therefore due to missing projections, whereas when it occurs twice at a spacing corresponding to 180° (e.g. around projections 750 and 2250), it suggests that the difference is intrinsic to the object.

Some other outliers are visible (e.g. between projections 1700–2300). On inspection, these were due to a very slight overall increase in grey scale throughout the image, probably caused by a slight reduction in flux. The filter applied during filtered backprojection reconstruction suppresses changes in brightness since it is a ramp filter in frequency space and so removes the DC component. Furthermore, this method was compared to two other methods of identifying missing projections: analysing the time-stamp of the image files and by tracking a known object in the projections. All three methods have advantages and disadvantages but largely agreed on the indices of the missing projections.

Fig 3 shows a cluster of outliers around projection 950. To determine the number of missing projections, sinograms at the level of a single well-defined feature in the fragment were generated specifically around the indices where some projections were identified as missing. A step could be seen in the sinogram where this occurred. An example is shown in Fig 2A, where clear discontinuities are visible corresponding around projection number 950. Because the sinogram was generated around a single, clear feature, it would be expected to be smooth. At each discontinuity, the number of missing projections, which needed to be added in order to space out the projections in the sinogram so that it became smooth, was found by visual inspection. The total number of missing projections was 25 and they came at ten different indices between projections 936 and 947. Fig 2B shows the sinogram after blank projections were added in place of the missing projections.

A fuller description of the methods used can be found in S1 Note: X-ray Techniques.

### Correcting image reconstruction

The basic problem was not so much the individual missing projections but the fact that the calculated angles of the projections after the problem segment were inconsistent with the angles of the projections before the problem segment. Standard CT reconstruction relies on the CT projections being evenly spaced around the object. Although reconstruction algorithms for unevenly spaced projections exist [12,13], we had a strong preference for using X-Tek’s standard image reconstruction software which has been optimised for their system.

Two strategies for correcting for the missing projections were tested. First, uniform projections with a uniform grey value of 64,000, equal to the zero-attenuation background value, were created and inserted, and second, the missing projection or projections were predicted by interpolation using a function written in Matlab that calculated the mean of two images either side of the missing projection. Where more than one projection was missing, the mean was weighted depending on how close the missing projection was to the ones on either side. In practice, it did not matter greatly how the missing projections were replaced: the important factor was to correct the angles of the projections after the problem segment.

## Results

### New CT reconstructions

Twenty-five missing projections were identified from 10 separate locations as the fragment rotated. Between one and nine projections were missing from each location. By compensating for these missing projections such that the angular locations of the remaining projections were correct, the images could be reconstructed with improved confidence. The full 3D reconstructed dataset is up to 32 GB (depending on the output resolution chosen) and is stored as a binary 3D array of floating-point numbers which can be viewed using various programmes. For analysis, 2D slices were identified by visual examination in Avizo lite Version 9.4. (ThermoScientific, USA) and analysed in Matlab v2016a.

**Fig 4:**
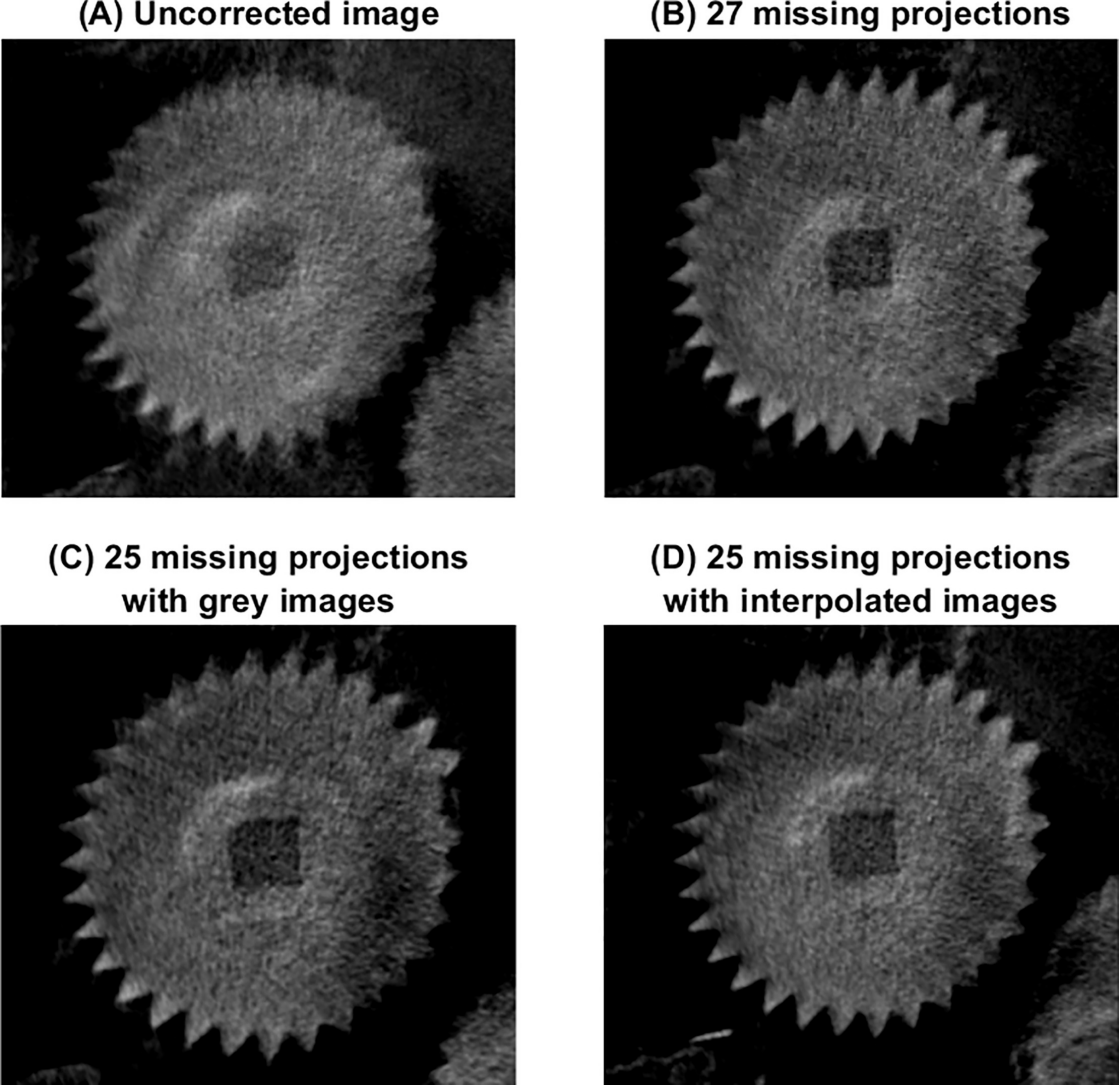
A magnified image of part of fragment A showing gear wheel b3, reconstructed using four methods to compensate for the missing projections. Fig 4A does not correct for missing projections; Fig 4B erroneously assumes 27 missing projections as had been previously assumed; Fig 4C correctly takes 25 missing projections and replaces the missing projections with grey images with a value of 64,000; Fig 4D uses a weighed mean of the projections on either side of the missing projection.

Fig 4 shows the same partial slice through a series of gear teeth for four images. The gear shown is Gear b3 which takes the output from the system of gears that calculates the lunar anomaly and reverses its direction of rotation so that the pointer will turn clockwise on the Zodiac Dial. Fig 4A shows the image reconstructed without correcting for the missing projections. Fig 4B is reconstructed assuming 27 missing projections as in previously published work ([5], [6]). Fig 4C takes the improved determination of 25 missing projections and added grey projections where the original projections were missing. Fig 4D uses a weighted mean of the projections on either side of the missing projection to interpolate a best guess as to the missing projection.

Visual examination of Fig 4 shows that the uncorrected image (Fig 4A) appears to have lower spatial resolution and lower contrast than Fig 4B, 4C and 4D which look similar to each other. This suggests that most of the deterioration in Fig 4A is due to the image reconstruction algorithm incorrectly assuming that the projections are evenly spaced around the object. The missing projections occur around projection 950, around a third of the way through the scan, meaning that the angles of all the final two-thirds of the projections are not consistent with the first third of the projections. Once the projections are spaced approximately correctly as in Fig 4B–4D, subsequent improvements can be seen but are modest. Quantitative analysis was unsatisfactory as it was very dependent on the precise geometry of the selected slices which could not be guaranteed to be identical across the different reconstructions.

The images reconstructed after correcting for the missing projections all displayed superior spatial resolution and contrast compared to the uncorrected images. However, there was little difference between the different methods used for correction. The decision was made, therefore, to analyse the images reconstructed after inserting grey projections as this method cannot bias the images by introducing additional information.

## Enhanced legibility of text

About 3,000 characters have been transcribed out of an estimated 15,000 characters originally present on the Mechanism [14]. These have been identified and interpreted using photography, reflectance transform imaging and previous X-ray CT reconstructions [5,6]. Each character is on average 1.6 mm high [6]. The combination of methods and the context have allowed the text to be interpreted with some precision, and the improved image reconstruction that we present here has allowed some of the previously uncertain text to be clarified.

**Fig 5:**
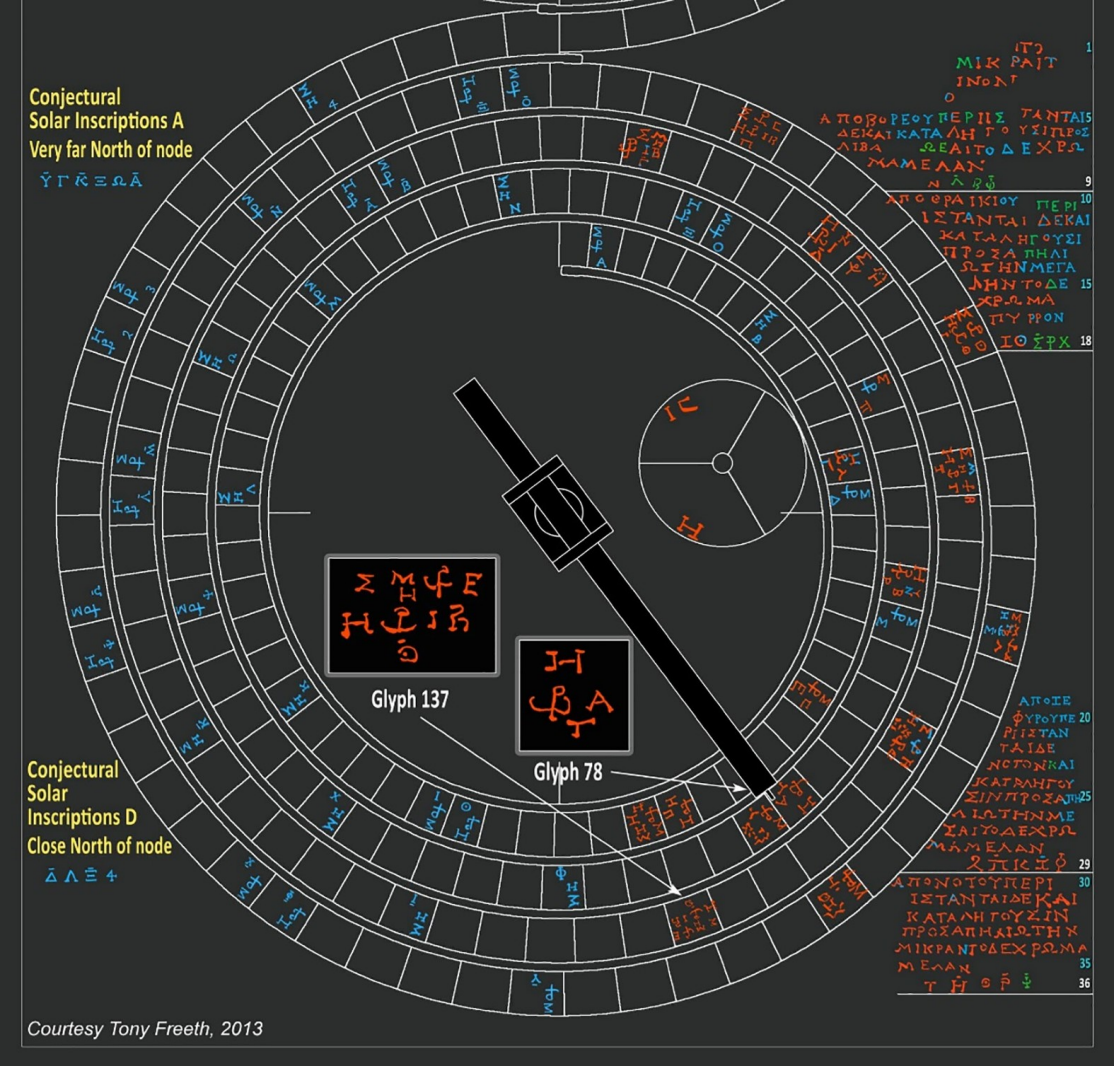
The 223-lunar month Saros Dial. Red text is traced from data; blue reconstructed from context; green is uncertain. Reproduced with permission from [6].

The Lower Back Dial of the Antikythera Mechanism is an eclipse prediction dial, based on the Saros Cycle of 223 lunar months [4–6]. Eclipse predictions are indicated by glyphs in the month cells of the dial, which include information about the eclipses: for example, whether the eclipse is solar or lunar and the time of the eclipse. Two examples are shown in Fig 5. Each glyph also includes an index letter and these refer to inscriptions round the Saros Dial. The inscriptions that survive can be seen on the right-hand side in Fig 5. All these refer to solar eclipses.

The index letters are grouped together in the eclipse inscriptions in Index Letter Groups, with around five index letters per group. These are also shown on the right-hand side in Fig 5, underlined in white. The lines of text immediately above these groups describe additional characteristics of the eclipses, such as colour and magnitude, which are shared by all the eclipses referred to in each Index Letter Group [6].

The anchor-like symbol in each glyph ω\^ρ^ is a ligature of ω and ρ, standing for ωρα meaning hour. The time of each eclipse follows this symbol and is indicated as the hour of the day, using the ancient Greek letters-for-numbers system (1 = A, 2 = B, 3 = Γ, … with an additional special symbol ϛ for the number 6 [15]). The hour data has profound consequences on the estimation of the eclipse times in Glyphs 13 and 125 [6]. The new corrected CT reconstruction has allowed some characters to be read with more confidence, which has settled these eclipse times. These data also directly resolve the index letter of Glyph 13, which was previously inferred indirectly from its context.

**Fig 6:**
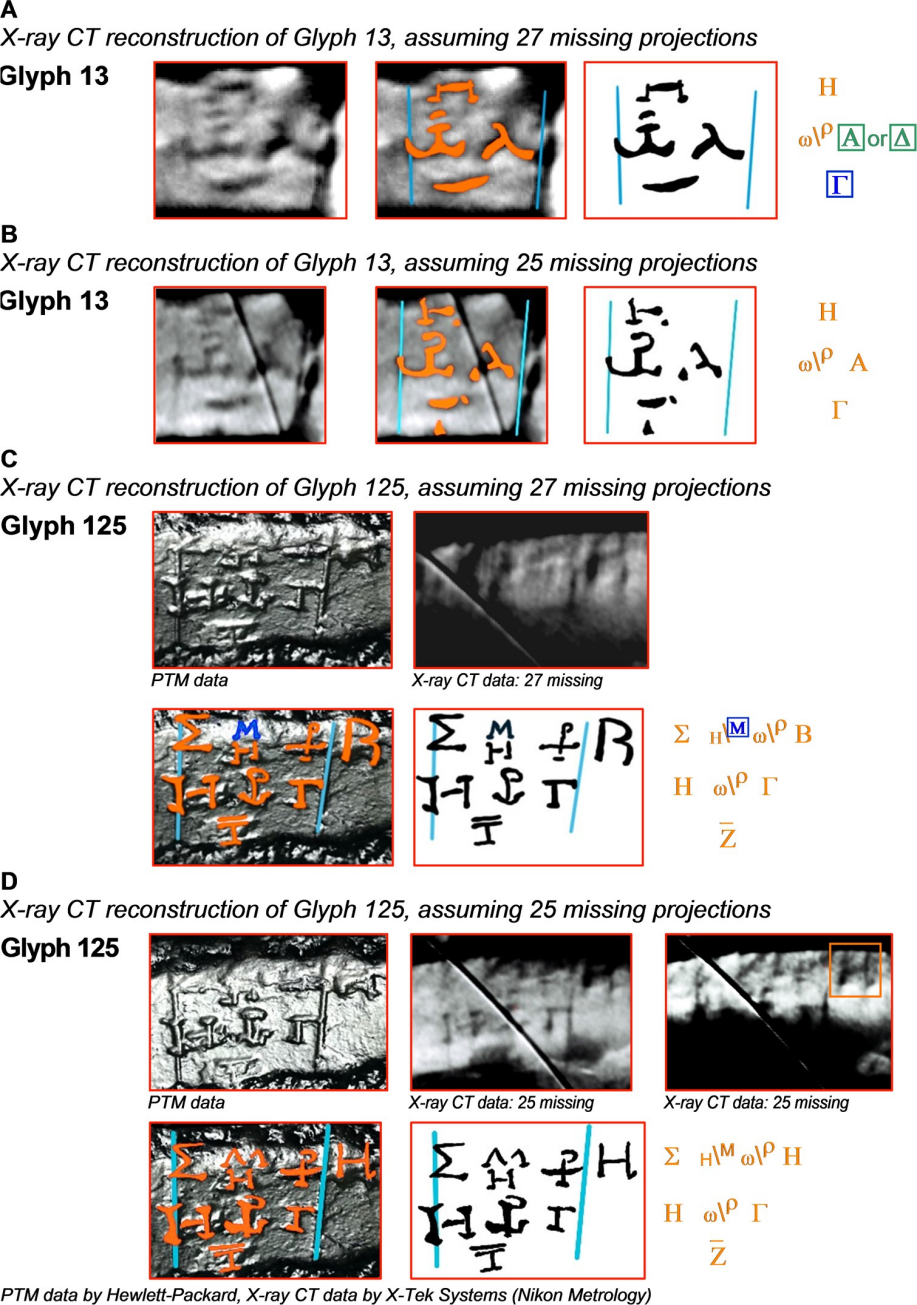
Deciphering eclipse times for Glyphs 13 and 125 on the Saros Dial. The X-ray CT images were reconstructed assuming 27 and 25 missing projections. Annotations shown in orange were traced from the data; those in blue (highlighted with a box) were inferred from the data or context and those in green (with a box) are of uncertain interpretation. The diagonal lines in some of the X-ray CT images are the result of dead pixels in the detector.

Fig 6 shows how the new X-ray CT reconstruction can significantly enhance the interpretation of the text. In Fig 6B, the solar eclipse time for Glyph 13 can now be read unambiguously as A, whereas previously (Fig 6A) it could have been either A or Δ [6].

When first published [5], the lunar eclipse time for Glyph 125 was interpreted as H (representing number 8) on the basis of a difficult reading of the reflection transform images. A subsequent publication [6] then re-interpreted the time as B (2), based on an indistinct X-ray CT image, shown in Fig 6C. Our new X-ray CT reconstruction of Fragment A now gives us a definitive answer. For Glyph 125, the left-hand of the two X-ray images in Fig 6D appears to be consistent with the old reconstruction—apparently showing B (2) as the eclipse time [6]. However, the surface of the plate is very uneven here and, by carefully angling the plane of the CT slice for the area of the plate showing the lunar eclipse time, it is now clear from the new X-ray CT volume reconstruction that the time is H (8) not B (2). The existence of the serif at the bottom of the right-hand stroke of the H (highlighted with an orange box in the right-hand image in Fig 6D) is definitive.

## Discussion

During CT image acquisition, 25 projections were lost from 10 different angular locations. This is most likely due to an intermittent problem with the communication link between the *BladeRunner* CT system and the acquisition computer. We have identified these missing projections and corrected for them, leading to a substantial improvement in images of the highest resolution scan of the largest fragment of the Mechanism compared to uncorrected images and a modest improvement compared to previously published images. We have also shown that, contrary to the literature, there was no movement of the fragment during imaging.

We have analysed images of the eclipse prediction dial and been able to confirm some uncertain characters and correct others.

## Supporting information

S1 Note X-ray techniques. (DOCX)
