# Supplementary material for: Improved X-ray computed tomography reconstruction of the largest fragment of the Antikythera Mechanism, an ancient Greek astronomical calculator
Source: PLoS One. 2018 Nov 9;13(11):e0207430. doi: 10.1371/journal.pone.0207430 (PMC6226198; doi:10.1371/journal.pone.0207430)
Supplement: S1 Note — (DOCX) [file pone.0207430.s001.docx]

# Note S1: X-ray Techniques

## X-ray computed tomography

The X-ray CT carried out by X-Tek Systems on the fragments of the Antikythera Mechanism employed a prototype X-ray machine, the *Bladerunner*. Fragment A, which is the subject of this study, is about 18 cm × 16 cm × 5 cm. When the X-ray study was proposed, the X-ray densities of the Antikythera fragments were not known. For example, it might have been possible that Fragment A, was predominantly composed of metallic bronze. X-ray CT requires that the sample be penetrated at all angles, including the “long axis” of the sample, which was about 16 cm in the orientation chosen for CT scanning. The *Bladerunner* was designed to use an X-ray tube voltage of 450 kV, which would be expected to penetrate the fragment whatever its composition. It turned out that the surviving fragments of the Antikythera Mechanism contain very little free metal: they are largely composed of corrosion products, calcification and sea accretions [4]. So, the full enhanced power of X-Tek’s prototype X-ray machine was not needed for the data gathering operation.

The *Bladerunner* acquires a series of planar X-ray images (known as “projections”; see Fig  1A) as the object rotates relative to the X-ray source and detector. The *BladeRunner* is a self-contained cabinet in which the source and detector are stationary and the object rotates. The projections were recorded on a Perkin Elmer flat panel detector.

## A Scan 6

*A Scan 6* was carried out on 17^th^ October 2005. The set-up used a 225 kV close focus X-ray head and a Perkin Elmer amorphous silicon flat panel detector. Many projections are required; scan 6 of Fragment A consists of 3052 projections. In X‑Tek’s software, the number of projections was not defined. Instead, a target number of projections was given, together with the time to acquire a full acquisition and the exposure time per image. However, uncertainties in the rotation speed of the object and delays in computer communication mean that the actual number of projections varied. Furthermore, the software oversampled the object by acquiring projections over more than a full rotation of the object. Each projection is saved as a 2048 × 2048 pixel 16-bit uncompressed TIFF image. This raw data was numbered proj0001 to proj3052 by the acquisition computer.

The projections were gathered while the sample rotated continuously. The reason for this is that the Antikythera fragments are extremely fragile. The research team were advised by X-Tek Systems that, if the X-ray machine were programmed to stop and start again for over 3000 projections, the resultant vibration might damage the fragments. The research team followed the advice from X-Tek Systems to ensure the absolute safety of the fragments.

Each projection image *P(x,y)* is corrected using dark and bright field reference images (*P_D_(x.y)* and *P_B_(x.y)*), acquired using an average of many images in order to reduce noise. The corrected image *P_C_(x,y)* consists of an array of corrected 16-bit grey values which are given by $P_{C}\left( x,y \right)=64,000\times\frac{P\left( x.y \right)-P_{D}(x,y)}{P_{B}\left( x,y \right)-P_{D}(x,y)}$ , so that a grey value of 64,000 in the corrected image is arbitrarily chosen to represent zero attenuation of the X-rays, and a grey value of zero represents complete (infinite) attenuation. These corrected images are fed into an implementation of the well-known Feldkamp, Davis and Kress (FDK) filtered backprojection algorithm [10], with the beam attenuation being calculated as log_e_(64,000 / *P_C_(x,y)*). The FDK algorithm filters then backprojects the values from every projection image through the volume, taking account of the inspection geometry and the projection angle, to calculate the X-ray linear attenuation values at the position of each volume element (voxel) within the reconstructed volume. These constitute the (floating-point) grey values within the CT volume. Specifically, images were reconstructed using Nikon CTPro3D version XT5.1.3.1 (Nikon Metrology, Tring, UK) while data and image analysis were performed in Matlab v2016a (The Mathworks, MA, USA).

## Movement of the fragment during the scan

Soon after the data were gathered, an initial volume reconstruction from the data was attempted by X-Tek Systems on the assumption that the scan data was all in order. This was unsuccessful in creating a meaningful volume: for example, features of the scan that should be circular had a helical appearance. It was hypothesised that the degraded reconstruction was because the object moved during the scan.

X-Tek’s protocol of oversampling the object meant that some projections were repeated at the beginning and end of the rotation. The oversampling meant that the full set of 3052 projections covered more than a single 360° rotation. A full rotation was identified by finding two similar projections that were 360° apart. A projection around 20 projections after the start of the scan was identified (to allow the X-ray machine to run up to speed). This was then compared with projections that were approximately 360° later in the scan. Since there were around 3000 projections, the incremental angle between projections was around 0.1°. It was found that projection number 2977 exactly matched projection 20, so proj0020 to proj2976 covered the full 360° rotation of the sample. These projections were then re-numbered (and corrected) as proj0001 to proj2957.

The difference between proj0020 and proj2976 was calculated and found to be no more than random noise, allowing the hypothesis that the fragment moved during the scan to be rejected.

## Missing Projections

After further research, it was realized that the basic problem was that some of the projections were missing. By stepping through the projections in sequence, it was possible to determine a segment of data between proj0936 and proj0972 where a series of projections at irregular intervals was missing: for example, there might be a gap of six missing projections followed by another gap with one missing projection and then two missing projections etc.. It appears that the computer failed to record some projections in the problem segment.

As an attempt to discover exactly where the missing projections occurred, the “time stamps” of each projection were examined. These are generated by the Windows operating system in increments of 100 ns. Unfortunately, the gaps between these time stamps were very variable. For example, in the series proj0020 to proj0029, the gap between time stamps varied from 2030976 units to 8281088 units. So, the time stamps did not prove to be useful in identifying the missing projections. The irregular spacing of the timestamps does not reflect the regularity of the angular spacing of the projections: the latter are determined by the high accuracy of the rotation of the sample turntable, while the former are mostly determined by the timing of communications between the X-ray machine and the acquisition computer.

The determination of exactly where the missing projections occurred was aided by viewing the data as a sinogram (see Fig 1B). A single horizontal line through a projection represents a single slice through the object. To generate a sinogram, the same horizontal slice is identified in each projection and displayed vertically in sequence with the same slice taken from every other projection. The sinogram shows how a single horizontal slice through the object varies with angle. In Fig. 1, a discontinuity can be seen in the sinogram at around 120° due to the missing projections. Using this method, it was determined that there were no repeated projections. If these had occurred, they would have shown up in Fig 3, which illustrates the sinogram. Missing projections cause a downward discontinuity in the sinogram; duplicated projections would have shown up as a similar upward discontinuity: none were apparent.

Missing projections were identified using a two-step process. First, the steps in the sequence where missing projections occurred were identified from the sinogram, and second, the number of missing projections at each step was found. Given that the gap between projections was of the order of 0.1°, it was assumed that neighbouring projections should be similar to each other. Accordingly, each projection *P_i_* was subtracted from the next projection *P_i+1_* for 1 < *i* < 2957 (where 2957 projections comprise a complete 360° rotation). The mean and standard deviation of the pixels making up each difference image were found. The null hypothesis was that the difference images form a random field of Gaussian noise. If this is true, a single pixel would be taken to be significantly different from the mean (at *p* < 0.05) if its value exceeds the mean ± 1.96 standard deviations (see any statistics textbook, for example [11]). In this case, however, we need to account for multiple comparisons as there are many pixels. We use the Bonferroni correction [11], and divide the threshold *p*value by the total number of pixels to give a new threshold of *p*. The Bonferroni correction is known to be conservative, and in this case gave a threshold of 5.3 standard deviations. Fig 2 shows the percentage of pixels that exceed mean ± 5.3 standard deviations for the difference between adjacent projections, plotted against projection number. Across the 2957 projections, most difference images show <3% of pixels exceeding this threshold, the number changing as the fragment rotates (and showing a reflection about the midway point as the fragment rotates by 180°). However, in some areas, the number of outlying pixels substantially exceeds the background number, suggesting that the difference between neighbouring projections is greater than expected. Moreover, when this occurs once in the pattern (e.g. around proj0950), it is likely to be inherent in the data collection and therefore due to missing projections, whereas when it occurs twice at a spacing corresponding to 180° (e.g. around proj0750 and proj2250), it suggests that the difference is intrinsic to the object.

Some other outliers are visible (e.g. between proj1700-proj2300). On inspection, these were due to a very slight overall increase in grey scale throughout the image, probably caused by a slight reduction in flux. The filter applied during filtered backprojection reconstruction suppresses changes in brightness since it is a ramp filter in frequency space and so removes the DC component.

The statistics rely on some incorrect assumptions, such as the difference images forming a Gaussian random field, and that the Bonferroni test is appropriate for large numbers of multiple comparisons. We therefore compared these results to results obtained using different thresholds from 2 to 10 standard deviations and verified by visual inspection that this threshold appeared to correctly determine the locations of missing projections. Furthermore, this method was compared to two other methods of identifying missing projections: analysing the time-stamp of the image files and by tracking a known object in the projections. All three methods have advantages and disadvantages but largely agreed on the indices of the missing projections—though, as discussed earlier, the time-stamp data was a poor guide.

Fig 2 shows a cluster of outliers around proj0950. To determine the number of missing projections, sinograms at the level of a single well-defined feature in the fragment were generated specifically around the indices where some projections were identified as missing. A step could be seen in the sinogram where this occurred. An example is shown in Fig 3A, where clear discontinuities are visible corresponding around proj0950. Because the sinogram was generated around a single, clear feature, it would be expected to be smooth. At each discontinuity, the number of missing projections which needed to be added in order to space out the projections in the sinogram so that it became smooth was found by visual inspection. The total number of missing projections was 25 and they came at ten different indices between proj0936 and proj0947. Fig 3B shows the sinogram after blank projections were added in place of the missing projections. Fig 3 shows a magnified view of the sinogram: (A) steps due to missing projection and (B) the flattened sinogram after correction for missing projections.

## Correcting image reconstruction

The basic problem was not so much the individual missing projections but the fact that the calculated angles of the projections after the problem segment were inconsistent with the angles of the projections before the problem segment. Standard CT reconstruction relies on the CT projections being evenly spaced around the object. Although reconstruction algorithms for unevenly spaced projections exist [12,13], we had a strong preference for using X-Tek’s standard image reconstruction software which has been optimised for their system. The version used was X-Tek Systems CTPro3D, Version XT 5.1.3.1. It was therefore decided to replace the missing projections.

Two strategies for correcting for the missing projections are presented. First, uniform projections with a uniform grey value of 64,000, equal to the zero-attenuation background value, were created and inserted, and second, the missing projection or projections were predicted by interpolation using a function written in Matlab that calculated the mean of two images either side of the missing projection. Where more than one projection was missing, the mean was weighted depending on how close the missing projection was to the ones on either side.

## New CT reconstructions

Twenty-five missing projections were identified from 10 separate locations as the fragment rotated. Between one and nine projections were missing from each location. The total number of projections gathered in 360° was 2957. Adding in the 25 missing projections made a total of 2982 for the volume reconstruction. These were numbered appropriately and inserted at the correct places, with later projections re-numbered to create a continuous sequence of corrected projections from proj0001 to proj2982.

By compensating for the missing projections such that the angular locations of the remaining projections were correct, the images could be reconstructed with improved confidence. The full 3D reconstructed dataset is up to 32 GB (depending on the chosen output resolution) and is stored as a binary 3D array of floating-point numbers which can be viewed using various programmes.
